# Crosstalk between Different DNA Repair Pathways Contributes to Neurodegenerative Diseases

**DOI:** 10.3390/biology10020163

**Published:** 2021-02-19

**Authors:** Swapnil Gupta, Panpan You, Tanima SenGupta, Hilde Nilsen, Kulbhushan Sharma

**Affiliations:** 1 Department of Clinical Molecular Biology, University of Oslo, 0318 Oslo, Norway; swapnil.gupta@medisin.uio.no (S.G.); panpan.you@medisin.uio.no (P.Y.); tanima.sengupta@medisin.uio.no (T.S.); 2 Section of Clinical Molecular Biology (EpiGen), Akershus University Hospital, 1478 Lørenskog, Norway; 3 Department of Neurology, Akershus University Hospital, 1478 Lørenskog, Norway

**Keywords:** neurodegeneration, DNA damage response, oxidative stress, PARP, ALS, Alzheimer, Parkinson, cGAS-STING, neuroinflammation

## Abstract

**Simple Summary:**

Constant exposure to endogenous and environmental factors induces oxidative stress and DNA damage. Rare brain disorders caused by defects in DNA repair and DNA damage response (DDR) signaling establish that failure to process DNA damage may lead to neurodegeneration. In this review, we present mechanisms that link DDR with neurodegeneration in these disorders and discuss their relevance for common age-related neurodegenerative diseases (NDDs). Moreover, we highlight recent insight into the crosstalk between the DDR and other cellular processes known to be disturbed during NDDs.

**Abstract:**

Genomic integrity is maintained by DNA repair and the DNA damage response (DDR). Defects in certain DNA repair genes give rise to many rare progressive neurodegenerative diseases (NDDs), such as ocular motor ataxia, Huntington disease (HD), and spinocerebellar ataxias (SCA). Dysregulation or dysfunction of DDR is also proposed to contribute to more common NDDs, such as Parkinson’s disease (PD), Alzheimer’s disease (AD), and Amyotrophic Lateral Sclerosis (ALS). Here, we present mechanisms that link DDR with neurodegeneration in rare NDDs caused by defects in the DDR and discuss the relevance for more common age-related neurodegenerative diseases. Moreover, we highlight recent insight into the crosstalk between the DDR and other cellular processes known to be disturbed during NDDs. We compare the strengths and limitations of established model systems to model human NDDs, ranging from *C. elegans* and mouse models towards advanced stem cell-based 3D models.

## 1. Introduction

The basic unit of our body, the cell, constantly encounters stress in various forms. Even though the brain is generally protected from many environmental agents, some external and endogenous stress conditions damage the genetic material (DNA). Endogenous DNA damaging agents are by-products of normal cellular metabolism. As a consequence of a high rate of oxygen consumption and metabolic activity, brain cells sustain a high burden of reactive oxygen species (ROS) that attack the DNA by oxidizing its bases and backbone. Having the lowest oxidation potential, the DNA base guanine is highly susceptible to oxidation [1]. Thus, the major, and best studied, DNA oxidation product 8-oxo-7,8-dihydro-2′-deoxyguanosine (8-oxoG) is among the most frequently used biomarkers for oxidative stress [2]. Hydrolytic deamination is another source of DNA damage under physiological conditions, leading to the loss of DNA bases and base modifications [3,4].

Moreover, many environmental agents associated with neurotoxicity and the development of neurodegenerative diseases (NDDs) [5], such as heavy metals (Pb, Cd, As, Hg, Cu, Zn and Fe) and pesticides (1-methyl-4-phenyl-1,2,3,6-tetrahydropyridine (MPTP), paraquat, dieldrin, rotenone), induce DNA damage via ROS formation. Rotenone and MPTP, for example, uncouple the mitochondrial electron transfer chain by inhibiting mitochondrial complex I [6]. There are two major strategies to protect DNA: antioxidant defense and DNA repair. There is ample evidence that the former is crucial for neuronal health, and several clinical trials are aiming to boost or stimulate antioxidant capacity.

Ageing is one of the main risk factors for most neurodegenerative diseases, including Alzheimer’s disease (AD) and Parkinson’s disease (PD) [7,8]. Hallmarks of ageing include genomic instability, telomere attrition, epigenetic alterations, loss of proteostasis, deregulated nutrient-sensing, mitochondrial dysfunction, cellular senescence, stem cell exhaustion, and altered intercellular communication [9]. Most of these biological processes may directly or indirectly contribute to NDDs. For instance, progressive damage to both nuclear DNA and mitochondrial DNA (mtDNA) is associated with aging. Damaged nuclear DNA can also lead to the activation of nucleus to mitochondria (NM) signaling. NM signaling is also associated with oxidative stress (through ROS generation and accumulation), which may ultimately contribute towards NDD [10]. Although oxidative DNA damage is believed to promote the aging process and contribute to the pathogenesis of several NDDs [11], the neuroprotective function of DNA repair is less clear. Proof of concept that DNA repair is important for brain health is provided by the existence of several rare NDDs caused by defects in DNA repair or DNA damage response (DDR) signalling, e.g., ocular motor apraxia, Huntington disease (HD) and certain cerebellar and spinocerebellar ataxias (SCA) [12].

In general, DDR signaling is activated in response to DNA damage. Central enzymes coordinating downstream DDR signaling include Ataxia Telangiectasia mutated (ATM), Ataxia Telangiectasia mutated and Rad3-related (ATR), and DNA-dependent protein kinase (DNA-PK). The DDR is highly complex and interconnected, and the precise pathways activated depend on the type of DNA damage, cell type and cell cycle stage. In general, the purpose of the DDR is threefold: (i) to detect and repair DNA damage, (ii) to coordinate the cells’ responses to DNA damage via activating a complex cellular cascade, and (iii) to induce cell cycle arrest or apoptosis if needed (we refer to recent reviews [13]). This orchestration is achieved by a DNA damage sensor, that, after activation, induce a signal transduction cascade with direct targets that promote several components of the cellular response to DNA damage, e.g., ATM phosphorylates H2A.X variant histone (gH2AX) which is important for transcription rewiring and the recruitment of repair proteins to chromatin. Of the many ATM targets, p53 is a master regulator of the cell cycle, which, when phosphorylated, leads to G1 arrest [14] and transcriptional activation of pro-apoptotic proteins [15].

Here, we give an overview of the repertoire of DNA repair pathways implicated in NDDs. We will also discuss the crosstalk between different DNA repair pathways and their role in other cellular processes known to affect the progression of some common NDDs. Finally, we discuss model systems that can be used to bring us closer to understand the mechanistic role and possible therapeutic potential of DNA repair in age-related NDDs.

## 2. DNA Damage Response and Repair Mechanisms

**Figure 1:**
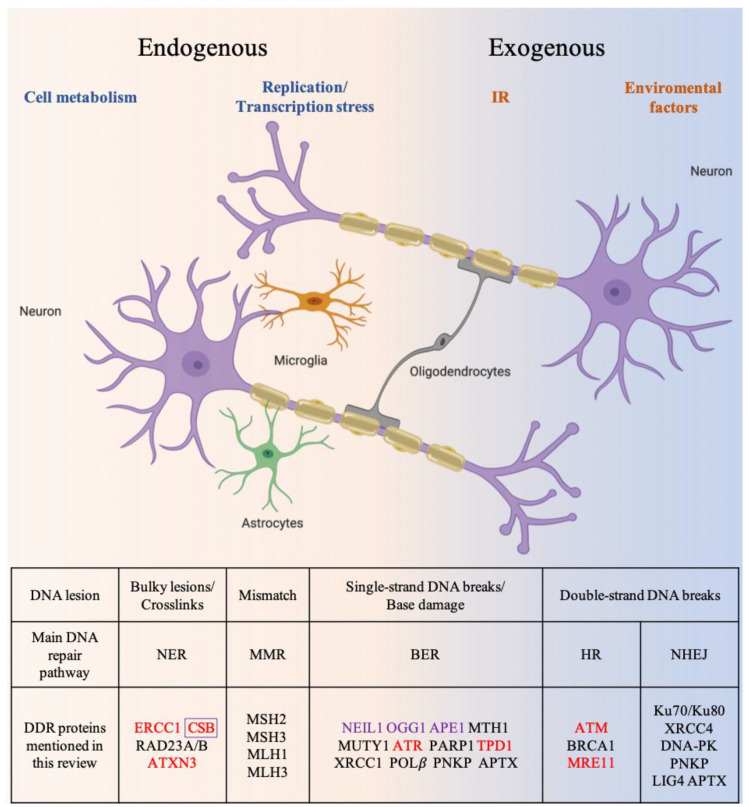
Overview of DNA damage and repair pathways. Classes of endogenous and exogenous DNA damage inducers in neurons and glia cells. Proteins involved in DNA damage response are shown under the heading of the main pathway they act or respond through. For a complete list of mitochondrial and nuclear DNA repair genes, readers are referred to recent comprehensive reviews [21,43]. A selection of proteins known or proposed to contribute to neurodegenerative disease (NDD) pathogenesis is highlighted in red. Selected proteins involved in mitochondrial DNA (mtDNA) damage repair are highlighted in purple. IR: ionizing radiation. Created with BioRender.com.

Mammalian cells are equipped with a highly elaborate DDR program that coordinates the cellular response to DNA damage [16]. Six major DNA repair mechanisms are integral to the DDR (Figure 1). Of these, direct reversal (DR), nucleotide excision repair (NER), base excision repair (BER), and mismatch repair (MMR) remove damaged and mismatched bases. Double-strand break repair (DSBR) pathways include non-homologous end joining (NHEJ) and homologous recombination (HR) [17]. NER is one of the most versatile DNA repair pathways and can handle a variety of lesions such as bulky DNA adducts formed by exposure to environmental genotoxic agents [18]. BER primarily corrects damaged DNA bases that do not create major structural distortions in DNA [19]. BER proteins are also important for the repair of single-strand breaks (SSBs). MMR corrects small insertion/deletion loops or mispaired nucleotides [20]. BER is considered to be the primary mechanism to counteract mitochondrial DNA damage [21]. The canonical NER, MMR and DSBR pathways do not operate in the mitochondria because many key enzymes are not localized in mitochondria. However, individual proteins are found in mitochondria, such as the NER proteins Cockayne Syndrome group B (CSB) and Xeroderma Pigmentosum Group D Protein (XPD) [22,23]. It remains to be demonstrated whether DNA repair pathways other than BER function in mitochondria, or other strategies are used to ensure mitochondrial DNA maintenance [24]. HR repairs double-strand breaks (DSBs) and inter-strand DNA crosslinks. Because HR requires a sister chromatid as a template for repair, it is restricted to S and G2 phases of the cell cycle, and is thus considered a “faithful DNA repair pathway”. NHEJ, in contrast, does not require homology and can directly seal blunt-ended breaks. Thus, NHEJ is active throughout the cell cycle [25,26]. As NHEJ does not utilize a template for repair and may involve end trimming to prepare ends for relegation [27], there is a risk that mutations are introduced. NHEJ is therefore referred to as “error-prone” [28]. Similar to nuclear DNA, mitochondrial DNA is also exposed to endogenous and exogenous agents.

In addition to DNA repair, DDR involves the activation of signaling pathways that coordinate the cellular responses to DNA damage (see [29] or [30] for a recent review). ATM, ATR, and DNA-PK orchestrate the downstream DDR signaling pathways by phosphorylating hundreds of enzymes [13]. Both ATM and DNA-PK catalytic subunits (DNA-PKcs) are activated in response to DNA double-strand breaks (DSB) [13] through different DNA damage sensor complexes. After activation by the MRE11-RAD50-NBS1 (MRN) complex, ATM phosphorylates several downstream DDR proteins, as described above [31,32,33,34,35]. However, ATM also activates several proteins that function in other cellular pathways. For example, ATM is also a regulator of mitophagy, oxidative stress responses and insulin signaling [36,37]. DNA-PKcs is activated by the Ku70/Ku80-DNA complex and is required for NHEJ [13,38]. Through its participation in NHEJ, DNA-PKcs plays a role in lymphocyte and neuronal differentiation, and also functions in post-integrational DNA repair in the case of human immunodeficiency virus-1 (HIV-1) infection [38,39,40]. During lymphocyte development, DSBs are generated in immunoglobulin and T cell receptor loci to generate immune-receptor diversity by V(D)J and class-switch recombination. DNA-PK mediated NHEJ is required to repair these DSBs for joining V(D)J recombination intermediates [39]. NHEJ factors (including DNA-PK) also play important roles in repairing DSBs that arise during the differentiation of neural progenitor cells. Specific mutations in DNA-PKcs affect NHEJ repair, and thus could lead to profound neurological defects [40,41]. ATR is closely related to ATM and DNA-PKcs [13]. ATR is activated by long stretches of single-stranded DNA coated by Replication Protein A (RPA) and many types of genomic stress, including stalled replication fork and transcription. Thus, in addition to its role in activating DNA damage checkpoint, ATR also functions in unperturbed DNA replication [42].

## 3. NDDs Related to the Defective DNA Repair Mechanisms

Defects in DNA repair proteins cause several rare diseases with neurodegeneration as part of the clinical phenotype. These rare disorders establish the general concept that neurodegeneration may result from defects in DNA repair. For instance, mutations in *APTX*, *PNKP* or *XRCC1* (all single-strand break repair (SSBR) genes) are linked to ocular motor apraxia [12], whereas defects in TDP1 (a SSBR gene) can lead to SCA with axonal neuropathy [44]. Similarly, mutations in the DSBR genes *MRE11* and the main DNA damage sensors proteins, ATM and ATR, can lead to cerebellar ataxia [13,45].

Moreover, DNA repair is involved in regulating the length of CAG repeats in some trinucleotide repeat disorders, such as HD and certain SCAs [46]. Genome Wide Association Studies (GWAS) in 4000 HD patients have shown a number of variants in DDR genes, particularly in MMR genes [47]. Much remains to be clarified concerning the mechanisms involved, but MMR and BER proteins have been suggested to generate the nicked DNA intermediate that serves as a template for expansion [48,49]. This is supported by CAG repeat expansion being abrogated in MMR-defective (e.g., *Msh2*, *Msh3*, *Mlh1* or *Mlh3* knockout) or BER-defective (e.g., *Ogg1* or *Neil1* knockout) mouse models of HD. The attenuation of somatic or germline repeat expansion was accompanied by amelioration of the HD-like phenotypes in some cases [50], which further supports a direct role of DNA repair enzymes in regulating CAG-repeat stability. Conversely, it has been proposed that CAG-repeat containing proteins, in which repeat expansion causes neurological disorders, function in DNA repair: examples include Huntingtin protein (HTT), the product of the huntingtin gene mutated in HD, which is recruited by ATM to sites of DNA damage [51]. Similarly, ATXN3, causing spinocerebellar ataxia 3 (SCA3), interacts with RAD23A/B, which are important players in NER [51]. Mutant ATXN3 has also been found to sequester PNKP outside the nucleus and thereby impair its ability to take part in DNA repair [52].

Recently, another indirect link between DNA repair and NDDs was shown in Spinal Motor Atrophy (SMA) disease, which is caused by deletion or mutation in the *survival of motor neuron1* (*SMN1*) gene [53]. The disease occurs in early childhood, causing motor neuron degeneration leading to muscular atrophy, asymmetric limb paralysis and death [53]. In SMA patient primary fibroblasts and non-dividing SMA neurons, a low level of SMN leads to DNA-PKcs deficiency, which impairs NHEJ and causes the accumulation of R-loops, DNA damage and neurodegeneration [54]. Conversely, increased gene expression of *survival of motor neuron2* (*SMN2*, a second copy of *SMN1*) [54,55] and SMN1 ectopic expression reduce R-loops, restore DNA-PKcs level and enhance NHEJ mediated DNA repair with reduced neuronal degeneration [54,55].

**Figure 2:**
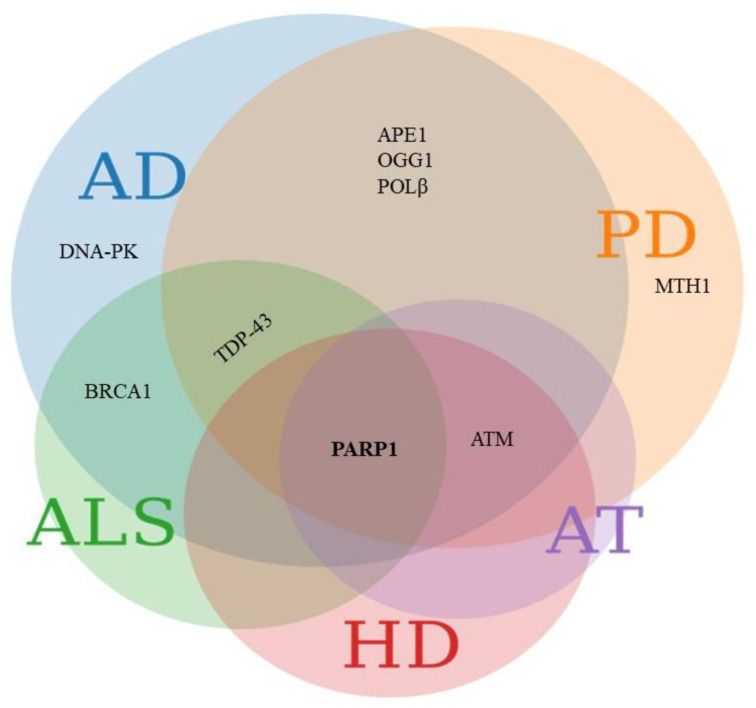
Common DNA damage response (DDR) proteins involved in various NDDs. The Venn diagram represents DDR proteins linked with various NDDs. Proteins involved in Alzheimer’s disease (AD) are depicted in the blue circle, Parkinson’s disease (PD) in orange, amyotrophic lateral sclerosis (ALS) in green, Huntington disease (HD) in pink and ataxia telangiectasia (AT) in purple. APE1, OGG1 and POLβ are involved in both AD and PD. Pathology related to the ALS associated protein TDP-43 is seen in both AD and PD. BRCA1 is involved in ALS and AD. Ataxia Telangiectasia mutated (ATM) is dysregulated in AD, PD, HD and AT. Poly [ADP-ribose] polymerase 1 (PARP1) emerges as commonly implicated in AD, PD, ALS, HD and AT. The figure was created using meta chart software.

These and similar reports establish that DNA repair pathways may contribute directly or indirectly towards NDDs. A possible causative role for DNA damage and DNA repair in the pathogenesis of more common, late onset NDDs is less well established, although DDR defects are emerging as possible culprits in diseases such as AD, PD and amyotrophic lateral sclerosis (ALS) (Figure 2).

### 3.1. Alzheimer’s Disease

Alzheimer’s disease (AD) is the most common age-related neurodegenerative disease, responsible for 60–70% of dementia cases worldwide [56]. AD is characterized by progressive impairment in cognitive function, impaired decision-making ability, behavioral disturbances, and gradual memory loss leading to dementia [57]. The disease presents two major neuropathological hallmarks—extracellular Aβ, deposited as neuritic or amyloid plaques, and neurofibrillary tangles (NFT), comprising highly aggregated hyperphosphorylated TAU [57].

AD is considered to be a multifactorial disease which involves complex interactions between intrinsic (ageing, genetics) and extrinsic (lifestyle, diet, environment) factors [58,59,60]. AD can be either familial, with mutations in the *APP*, *APOE*, *PSEN1* and *PSEN2* genes [61,62,63], or sporadic, which accounts for more than 90% of cases. Sporadic AD is a polygenic disease [64] with a strong contribution of *APOE* [65,66].

Several types of DNA damage, e.g., DSB, SSB and 8-oxoG, are detected in AD brains [67,68,69,70,71]. The reduced expression and activity of many DDR proteins (ATM, BRCA1 and DNA-PK) have been associated with AD pathology [72,73,74]. Similarly, the expression of BER genes, such as *OGG1* and *NEIL1*, is reduced in AD [75,76,77]. Another BER gene, DNA Polymerase β (*POLβ*), whose expression is reduced during senescence and aging [78,79], is also suggested to play some role in AD, as a reduction in DNA-gap filling activity in mild cognitive impairment (MCI) and AD brains was accompanied by the decreased expression of *POLβ* [76]. Consistently, transgenic AD mice with genetic downregulation of *Polβ* (*Polβ^+/−^)* showed impaired memory and synaptic plasticity, increased neuronal death, and DNA damage accumulation [80]. All these studies indicate that there is a correlation between AD and inefficient DNA repair; however, further studies are required to establish a direct causal link between the two.

### 3.2. Parkinson’s Disease

Parkinson’s disease (PD) is the second most common neurodegenerative disorder, affecting 1% of the population over 60 years of age worldwide. The disease is characterized by asymmetrical bradykinesia, rigidity, resting tremor, and postural instability. The cardinal pathologic features of PD include the progressive loss of dopaminergic neurons in the pars compacta of substantia nigra (SN), α-synuclein (α-SYN) aggregation, and Lewy body formation in the mid-brain region. Like AD, PD is a multifactorial disease caused by both environmental and genetic factors. Familial PD is associated with mutations in *LRRK2*, *PARK7*, *PINK1*, or *SNCA* [81,82].

Since oxidative stress and mitochondrial dysfunction are prominent features in PD, there has been a focus on studying the neuroprotective properties of mitochondrial DNA repair. Indeed, recent genetic data suggested that, on a pathway level, variants in genes involved in mtDNA maintenance may be enriched in PD patients [83]. The role for nuclear DNA repair is less well established, but elevated levels of 8-oxoG, SSBs and DSBs have been found in both PD post mortem human brains and in rat PD models [84,85,86]. Mice heterozygous for a deletion in the NER gene *Ercc1*, which only functions in nuclear DNA repair, had increased levels of phosphorylated α-SYN, γH2AX foci in the striatum, and developed severe PD-like pathology following MPTP administration [87]. These findings support that the nuclear DNA repair machinery plays a role in PD pathology, but the mechanisms involved remain elusive.

The BER DNA glycosylases OGG1, MTH1 and MUTY1 are highly expressed in SN and associated dopaminergic neurons in PD brains [88,89,90]. Consistently, *Ogg1* knock-out mice showed behavioral defects and elevated 8-oxoG levels [90,91]. A direct, functional coupling between PD susceptibility genes and BER is suggested from the observation that Parkin ubiquitinates Apurinic/apyrimidinic endonuclease 1 (APE1) under stress. Moreover, mutations in *PRKN* abrogate APE1 ubiquitination, resulting in the overactivation of APE1 and resulting in SSBs formation [92]. Elevated levels of AP sites were found in mtDNA in nigra l dopaminergic neurons from PD patients [93]. Polymorphisms in *APE1* and *OGG1* have been suggested to increase the risk of PD [94,95], but no individual single nucleotide polymorphism (SNP) in BER genes is consistently found to be associated with PD risk across cohorts [96,97]. However, elevated levels of pathological α-SYN correlated with increased PARP1 levels and PARylation in the brain and cerebrospinal fluid of PD patients, supporting the activation of cellular responses to SSBs. Interestingly, nuclear α-SYN may bind DNA and DDR proteins [98], and increased PARylation has been shown to promote pathological α-SYN aggregates [99]. Taken together, evidence of a direct functional coupling between DDR and α-SYN is emerging, suggesting a potential role for nuclear DDR in proteotoxicity in PD.

### 3.3. Amyotrophic Lateral Sclerosis

Amyotrophic lateral sclerosis (ALS) is an adult onset degenerative motor neuron disease, affecting the motor cortex, brain stem and spinal cord, leading to progressive paralysis and eventually death [100]. ALS is considered, primarily, a sporadic disease, but about 10% of ALS cases are familial [101]. More than 40 genes are currently linked to ALS, and the majority of cases, both familial (*f*ALS) and sporadic ALS (*s*ALS), are caused by mutations in four genes: *C9ORF72*, *SOD1*, *TARDBP* and *FUS/TLS* [102,103]. Over 30 years ago, a hypothesis suggesting the accumulation of abnormal DNA as the primary abnormality in ALS was brought forward [104]. This has later been substantiated by many studies showing AP sites, DNA SSBs and 8-oxoG accumulation in diseased human motor neurons [105,106], further supporting that defective DDR is involved in ALS.

Defects in *SOD1*, the first identified ALS gene, which acts as a scavenger against free radicals, link oxidative damage with ALS [107]. The activation of several key proteins that function in sensing DNA damage, e.g., ATM, has been shown in human ALS motor neurons with SOD1 mutations, suggesting DNA damage accumulation in SOD1 deficient neurons [105]. Whole exome sequencing revealed NIMA-related kinase 1 (*NEK1*) as another ALS associated gene. NEK1 functions in cell-cycle checkpoint control [108] and contributes to DDR independent of ATM and ATR [109]. Human induced-pluripotent stem cells (iPSCs) and differentiated motor neurons carrying *NEK1* mutations show dysregulation of the DDR machinery and increased DNA damage [110].

APE1 and OGG1 are upregulated in ALS brains [111] and spinal cord motor neurons in *SOD1* transgenic mice, respectively, indicating increased DNA oxidative base damage [112]. Hypomethylation of the *APE1* and *OGG1* promoter regions has recently been described in ALS [105]. The authors speculated that hypomethylation might represent a compensatory upregulation of these BER genes in the vulnerable ALS neurons in order to cope with oxidative stress. Interestingly, two RNA/DNA binding proteins encoded by ALS pathogenic genes, *TARDBP* and *FUS/TLS*, function in DNA damage response. Mitra et al. showed DSBs accumulation and reduced recruitment of the XRCC4-XLF-DNA ligase 4 (LIG4) complex at DSB sites, thus indicating attenuated NHEJ in TDP-43 (encoded by *TARDBP*) depleted motor neurons. Moreover, the authors demonstrated that TDP-43 acts as a scaffold, facilitating the recruitment of the XRCC4/LIG4 complex to DSBs [113]. Recent studies revealed that FUS is recruited to DSBs and interacts directly with PARP1 [114,115]. Moreover, interaction between FUS and Histone deacetylase 1 (HDAC1) promotes NHEJ [116,117]. A non-canonical translation mechanism leads to the production of five dipeptide repeat proteins (DPRs) from the hexanucleotide repeat expanded *C9ORF72* gene. A recent study indicated that, among the five DPRs, proline-arginine (PR), glycine-arginine (GR), and glycine-alanine (GA) are the most neurotoxic and decrease the efficiency of NHEJ, single-strand annealing and microhomology-mediated end joining (MMEJ) [118]. Consistently, increased levels of several DDR markers (γH2AX, phosphorylated ATM, cleaved PARP1 and 53BP1) were observed in spinal cord motor neurons of *C9ORF72* ALS patients [119].

Taken together, these studies suggest that several DNA repair pathways are implicated in NDD pathogenesis; however, the exact molecular mechanisms by which the responses to genomic stress contribute to neurodegeneration are still unclear.

## 4. Crosstalk between DDR and Age-Related Neurodegenerative Diseases

From the above, it is clear that impaired DDR may contribute directly or indirectly towards various NDDs. Moreover, individual DDR proteins may be involved in more than one NDD (Figure 2). Dysregulation, dysfunction, or inactivation of ATM is reported in AD and PD. Using a fly model, Petersen et al. showed that even a slight reduction in ATM kinase activity caused neurodegenerative features [120]. Reduced ATM levels and activity have been shown in hippocampal and frontal cortex neurons in AD brains as well as in AD transgenic mice [72]. The dysregulation of ATM signaling was also reported in HD where, contrary to AD or AT, increased or persistent activation of ATM signaling correlated with disease progression [121,122]. In a rodent PD model, α-synucleinopathy led to the upregulation of *γ*H2AX, 53BP1 and the phosphorylation of ATM in neurons [87,123], suggesting that DSB might contribute to DA neurodegeneration in aging brains.

The DSB repair protein BRCA1 affected cognitive function in transgenic human amyloid precursor protein *(hAPP)* AD mice, where small-hairpin RNA mediated knockdown was accompanied by a reduction in memory and learning ability [73]. In post mortem AD brain, hypomethylation of the *BRCA1* promoter region was accompanied by the upregulation of expression and cytosolic mislocalization of BRCA1 [124]. In AD, the expression of BRCA1 was increased, possibly as a consequence of oxidative DNA damage accumulating due to Aβ-induced ROS formation [125]. However, in this case, the upregulation of BRCA1 did not translate to increased DDR capacity because BRCA1 remained dysfunctional and co-aggregated with TAU in a highly insoluble form, making it unavailable for DNA repair [124,126,127,128]. Hence, increased expression does not always lead to increased repair capacity, but it is suggested that BRCA1 can function by a dual mechanism in AD: while it can cause abnormal ubiquitination and sub-cellular distribution of presenilin 1 (PS1) and thereby affect Aβ processing, it can also induce pro-apoptotic signaling [128]. Hence, also in AD the emerging evidence suggests a direct functional coupling between DDR and central pathogenic components. Whether this is also the case in ALS remains to be determined, but transcriptomic profiling shows that BRCA1 is highly expressed in the microglia of human ALS patients [129].

Mice deficient in the NHEJ repair enzymes LIG4 and XRCC4 along with Ku70 and Ku80 show apoptosis of post mitotic neurons [130]. Abnormal expression of DNA-PK has been reported in AD. Reduced NHEJ activity in cortical neurons and cortical extracts from AD brains was ascribed to lower protein levels of DNA-PK and its regulatory subunit Ku80 [131,132]. Aβ aggregates inhibit DNA-PK activity in nerve growth factor-differentiated PC12 cells by reducing the expression of DNA-PKcs in a ROS dependent manner [133]. Aβ can enter the nucleus of PC12 cells and downregulate the expression of DNA-PKcs through a mechanism independent of oxidative stress, thus indicating that Aβ itself may attenuate DNA-PKcs activity and hence reduce NHEJ capacity. As mentioned above, DNA-PK deficiency also leads to SMA [55].

PARP1 is activated by SSB to synthesize poly-ADP ribose (PAR) polymers at the damaged site [134,135,136,137,138,139]. Constitutive PARP1 activation can deplete intracellular NAD^+^ levels, which can affect mitochondrial homeostasis, ROS production, DNA repair, and cell death [140,141,142]. A correlation between PARP1 activation and AD has been shown in AD human brains and AD mouse models [143,144,145,146]. Additionally, it has been shown that MPTP treated mice exhibit PARP1 activation [147]. A mechanistic link between PARP1 activation and neurodegeneration was supported by *Parp1^−/^^−^* mice being resistant to the toxic effects of MPTP [147]. In ALS patients, PARP1-mediated, caspase independent programmed cell death of motor neurons through parthanatos was reported in the spinal cord [148,149,150].

Thus, PARP1 emerges as one of the central DDR proteins involved in most of the NDDs (Figure 2). The available evidence suggests that PARP1 mediated AD and PD pathologies could result from several cellular pathways: (i) bioenergetic deficit via NAD^+^ depletion; (ii) the activation of apoptosis via interaction with tumor suppressor and apoptotic genes such as *TP53* and *BCL2*; (iii) the induction of parthanatos; and (iv) transcription rewiring via the modulation of transcriptional factors [145]. A causal role for PARP1 activation in NDD pathogenesis is further substantiated by studies where PARP1 inhibition and NAD^+^-augmentation prevented neuron degeneration in PD animal models [99,151]. Based on this, Nicotinamide riboside supplementation was proposed as a possible therapeutic intervention for NDDs [151].

## 5. Crosstalk between DDR and Other Cellular Processes Known to Be Disturbed in NDDs

**Figure 3:**
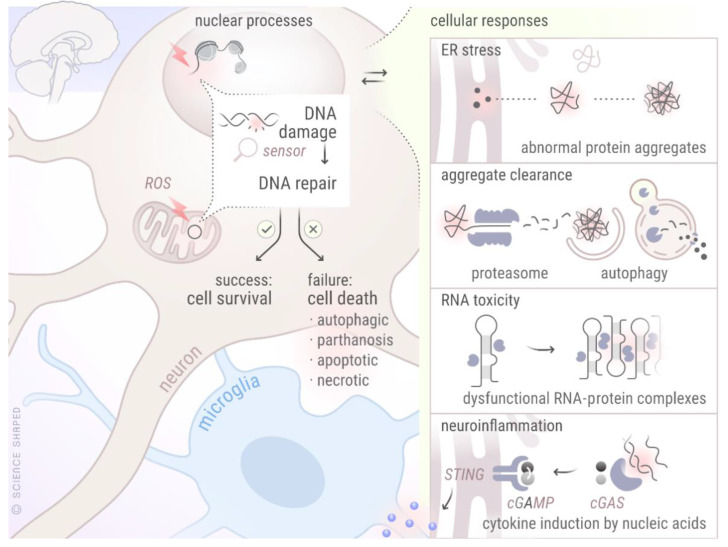
Crosstalk between different cellular mechanisms involved in various NDDs. The figure represents crosstalk between DDR and other cellular processes known to be perturbed in various NDDs. Created by Ellen Tenstad @ScienceShaped.

From the above, it is apparent that defects in DNA repair proteins may contribute towards the initiation and progression of more than one NDD. In this section, we will describe some of the unifying mechanisms observed in various NDDs and will provide the evidence for their crosstalk with the DDR machinery (Figure 3).

### 5.1. DDR and Autophagy

Inefficient aggregate clearance indicating defective autophagy is one of the common features of NDDs. During AD pathogenesis, both impaired autophagosome synthesis and reduced clearance of autophagic substrates have been shown [152,153,154]. The accumulation of α-SYN in PD indicates dysfunctional chaperone mediated autophagy (CMA), a major contributor for the autophagic degradation of α-SYN [155]. Mutations in many autophagy receptors, including SQSTM1/p62 [155], OPTN, and UBQLN2, have been associated with the pathogenesis of ALS [156,157,158]. Associations between DDR and autophagy as a direct cause of NDDs are not yet well-established, although the literature suggests indirect connections between the DNA repair machinery and autophagy through various, non-exclusive routes [159,160,161]. ATM activation can cause autophagy induction through 5′ AMP-activated protein kinase (AMPK) in response to ROS [162,163]. While *Atm^−/−^* neurons show abnormal autophagy, ATM itself is processed through autophagic degradation [164]. *Parp1* knockout mice subjected to acute starvation displayed deficient liver autophagy, implying a physiological role for PARP1 in starvation-induced autophagy [165]. The link between DDR and autophagy seems bidirectional, as knocking out BECLIN1, a protein involved in autophagy induction, reduced the activity and levels of crucial HR and NHEJ proteins, and also attenuated the formation of DNA-PK complexes [166,167]. SQSTM1/p62 has been shown to regulate the ratio between HR and NHEJ by promoting the latter upon radiation [168]. CMA regulates CHK1 levels and prevents the hyperphosphorylation and destabilization of the MRN complex [169]. The translation product of ultraviolet irradiation resistance-associated gene (*UVRAG*), an important player in autophagy initiation, patrols DSB repair activity through direct binding to DNA-PK in NHEJ [170]. Interestingly, DNA damage could induce mitophagy, which may play a role to protect cells against DNA-damaged induced cellular death [171]. Although not explored for DDR, our group recently showed the induction of endoplasmic stress (ER stress) mediated pro-survival autophagy after radiation induced ROS induction [172].

### 5.2. DDR and Neuroinflammation

Neuroinflammation is another common phenomenon noticed in different NDDs. A vicious cycle is believed to operate, involving neuroinflammation, oxidative stress, and neurodegeneration [173,174]. Inflammation can itself induce DNA damage through the generation of ROS and reactive nitrogen species (RNS). Inflammation increases not only mutagenic DNA lesions, such as 8-nitroguanine and 8-oxoG, but also negatively impacts on DNA repair capacity by inhibiting many important DNA repair enzymes [175,176]. The seminal discovery of cyclic guanosine monophosphate-adenosine monophosphate (GMP-AMP) synthase (cGAS) as the mammalian cytosolic DNA sensor by Sun et al. has established direct links between damaged DNA and neuroinflammation [177]. Upon binding to cytosolic DNA, cGAS converts ATP and GTP to cyclic GMP-AMP (cGAMP), which binds and activates the endoplasmic reticulum protein stimulator of interferon genes (STING), finally inducing the production of type I interferons through the transcription factors NF-kB and IRF3 [178]. The activation of cGAS-STING has been seen in several NDDs: in an *Atm*-deficient rat model with neuroinflammatory and neurodegenerative phenotypes, an accumulation of cytosolic DNA leading to enhanced levels of phosphorylated TBK1 (indicative of STING activation) was observed in various cell types, including microglia [179]. Xu et al. demonstrated that cGAMP can suppress AD by elevating TREM2 levels [180]. In iPSC derived ALS patient motor neurons, TDP-43 triggered the release of mtDNA, leading to cGAS-STING activation, and neuroinflammation has been shown [181]. The induction of IL6 in response to circulating cell-free mtDNA has been reported in Parkinson patients’ serum [182]. Hence, cGAS-STING mediated inflammation emerges as another common characteristic of NDDs.

In summary, individual DDR proteins can be involved in more than one NDD (Figure 2), and can induce neurodegenerative features through their interaction with various cellular processes (Figure 3).

## 6. Model Systems to Study NDDs and Therapeutics

Although post mortem brain tissue is useful to map major structural or molecular differences in DDR markers in late stages of NDDs, it has limited power to explain disease mechanisms. Thus, animal models remain essential tools to study the mechanisms driving neurodegeneration in a life-course perspective. A selection of studies linking DDR to NDDs using various model systems discussed above or suggesting appropriate models to study this crosstalk is summarized in Table 1. As is evident from the above discussion, rodents remain the most extensively used model animals. For AD, for example, there are more than two hundred different rodent models (https://www.alzforum.org/research-models/search (accessed on 19 February 2021)). However, no model recapitulates all cardinal phenotypes seen in the human disease, illustrating the need for better models to model human NDDs.

Invertebrate animals are relatively inexpensive to maintain and have a short lifespan and reproduction cycle. These properties make non-vertebrate models amenable for drug and genetic screening. The nematode *Caenorhabditis elegans* (*C. elegans*) is an example of an animal where its transparent body allows the monitoring of neurodegeneration in vivo. Humanized animal models are available in which transgenic animals express human α-SYN [187,188], Aβ_1–42_, TAU [189,190], TDP-43 and SOD1 [191,192,193]. The existence of behavioral assays makes it possible to study viability and functionality from individual neurons during a natural life course. Hence, simple animals offer excellent opportunities to study the combined effects of genetic and environmental risk factors in conjunction with aging, the most important risk factor for NDD. With respect to the contribution of DDR, *C. elegans* has less redundancy of DNA repair genes, making it a great tool to define whether and how DNA repair proteins affect neurodegeneration. A limitation of *C. elegans* is that it does not have a brain. Thus, vertebrate models are needed. Of these, *Danio rerio* (*D. rerio*) is increasingly popular as it has vasculature and a brain separated by a blood–brain barrier [194,195,196].

**Figure 4:**
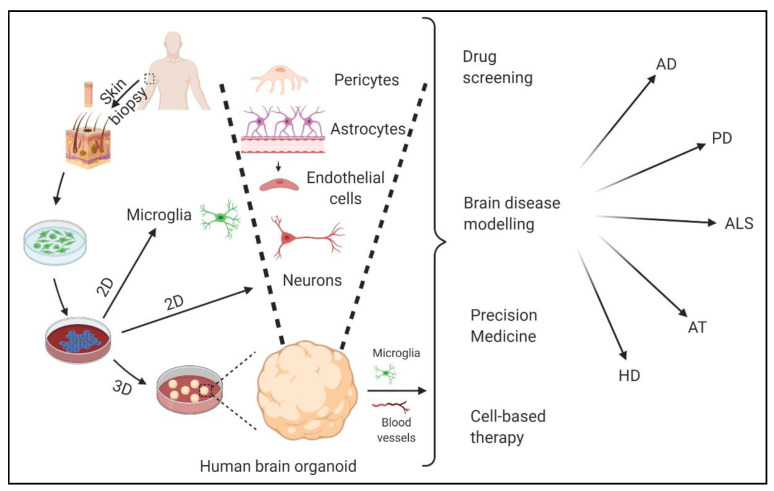
Potential of 2D iPSCs and 3D-organoids to models NDDs. Skin fibroblasts derived from NDD patients can be reprogrammed into iPSCs. These iPSCs can be differentiated into various brain cell types including different neuron types, microglia, etc. iPSC-derived 3D-organoids contain most of the brain cell types (except microglia and vasculature, which can be co-cultured with 3D-organoids) and are able to show major disease phenotypes. Various applications of these model systems have also been shown. Created with BioRender.com.

The development of human iPSC technology has opened the doors to modeling complex brain diseases. Established protocols are available to generate various brain cell types from patient derived iPSCs in two dimensional (2D) monolayer cultures and use these to model AD [197,198], ALS [199,200,201] and PD [202,203]. The possibility of introducing genome editing in iPSCs (e.g., via CRISPR/CAS9) further increases the potential of these cells to study the specific roles of individual genes. In addition, the role of other brain cell types (including glial cells, also derived from iPSCs) can be studied in recently developed co-culture systems [204]. Still, the 2D culture systems do not recapitulate the complexity of the brain, and phenotypes such as extracellular protein aggregation are difficult to observe. Thus, 2D iPSCs are not the most preferred choice to model NDDs. Self-organization of embryoid bodies from iPSCs, so-called 3D-organoid models, may allow us to study cellular development and inter-cellular interactions within a 3D human brain microenvironment [205]. Advances in 3D-culture systems have led to the formation of multiple brain cell types and specific brain regions in these organoids (mini-brains) [206]. Forebrain cortical organoids, mid-brain organoids, and motor neuron spheroids derived from AD and HD, PD and ALS patient iPSCs, respectively, are able to show the cardinal tissue phenotypes and can, thus, be very useful for understanding these complex NDDs (Figure 4) [207,208,209,210,211,212,213].

## 7. Conclusions

To summarize, both antioxidant defense and DNA repair prevent the accumulation of DNA damage and thereby counteract the development of NDDs. One example for antioxidant defense is the scavenger superoxide dismutase1 (SOD1) that protects neurons against amyloid β (Aβ) mediated neurotoxicity [214]. Both the overexpression of wild type SOD1 and mutations in SOD1 are associated with several age-related NDDs [11,215,216]. In fact, several clinical trials are targeting wild type SOD1 or mutant SOD1 to prevent aggregation and misfolding in NDDs, and an antisense oligonucleotide is now in a phase III clinical trial (NCT02623699). A small molecule, Arimoclomol, which promotes the proper folding of SOD1 in the endoplasmic reticulum (ER) [11], is in a phase III trial (NCT03491462). Whether targeting DNA repair or the DDR may be developed into therapeutic options against NDDs remains to be clarified. However, the emergence of PARP1 activation by SSB as a common finding in NDDs and the availability of PARP1 inhibitors suggest that this might be a path to pursue. However, directly targeting PARP1 carries risks of deleterious effects on other cells and tissue systems. One consequence of PARP1 overactivation is the depletion of cellular NAD^+^, which is a substrate of PARP1. In our previous work, we showed that boosting NAD^+^ levels is a promising strategy to stimulate DNA repair and prevent neurodegeneration in rare DNA repair diseases [140,146,184]. To further explore the therapeutic potential of DDR, cross-species studies where different model systems are combined to recapitulate different aspects of these complex diseases can be used. iPSC derived 3D-organoid models emerge as promising model systems that can capture the complexity of the DDR and its crosstalk with other cellular processes important to maintain neuronal health.

## Figures and Tables

**Table 1:** Different model systems to study NDDs and the involvement of DDR. The table shows various model systems that have been used to study the role of DDR in various NDDs. Xeroderma Pigmentosum, CS: Cockayne Syndrome.

Model	DDR Genes	NDD	References
*C. elegans*	*PARP1*	AD	[146]
	*TARDBP*	ALS	[183]
	*PARP1*	AT	[140]
	*XPA*	XP	[184]
	*CSB*	CS	[184]
	*ATM*	AT	[184]
*D. melanogaster*	*ATM*	AT	[72]
		AD	[120]
		HD	[122]
*D. rerio*	*C9ORF72*	ALS	[185]
	*ATM*	AT	[186]
*M. musculus*	*ATM*	HD	[122]
		AD	[72]
	*BRCA1*	ALS	[129]
		AD	[73]
	*POLβ*	AD	[80]
	*OGG1*	PD	[91]
		ALS	[112]
	*ERCC1*	PD	[87]
	*PARP1*	AD	[145]
		PD	[147]
		ALS	[150]
*R. norvegicus*	*ATM*	PD	[123]
iPSCs	*BRCA1*	AD	[128]
	*NEK1*	ALS	[110]

## Data Availability

No new data were created or analyzed in this study. Data sharing is not applicable to this article.
